# *Trichosporon austroamericanum* Infections among Hospitalized Patients, France, 2022–2024

**DOI:** 10.3201/eid3111.250503

**Published:** 2025-11

**Authors:** Emilie Burel, Catherine Sartor, Valérie Moal, Vincent Bossi, Jacques Sevestre, Justine Solignac, Rémi Charrel, Marie Desnos-Ollivier, Stéphane Ranque, Estelle Menu

**Affiliations:** Aix-Marseille Université, Marseille, France (E. Burel); Institut Méditerranée-Infection, Marseille (E. Burel, V. Bossi); Assistance Publique Hôpitaux de Marseille, Hôpital Conception, Equipe Opérationnelle d'Hygiène Hospitalière, Marseille (C. Sartor, R. Charrel); Aix Marseille Université, Institut de Recherche et Développement, Microbes Evolution Phylogeny and Infections, Marseille (V. Moal); Assistance Publique Hôpitaux de Marseille, Hôpital Conception, Centre de Néphrologie et Transplantation Rénale, Marseille (V. Moal, J. Solignac); UMR D257 RITMES, Aix-Marseille Université, Assistance Publique Hôpitaux de Marseille, Service de Santé des armées, Marseille (J. Sevestre, S. Ranque, E. Menu); Unité des Virus Émergents, Aix-Marseille Université, Università di Corsica, IRD 190, Inserm 1207, IRBA, Marseille (R. Charrel); Institut Pasteur, Université Paris Cité, National Reference Center for Invasive Mycoses & Antifungals, Mycology Translational Research Group, Paris, France (M. Desnos-Ollivier)

**Keywords:** Trichosporon austroamericanum, fungi, emerging disease, Trichosporonosis, yeast, opportunistic agents, outbreak, France

## Abstract

During 2022–2024, six cases of invasive fungal infection occurred among immunocompromised patients at Marseille University Hospital, Marseille, France. Matrix-assisted laser desorption/ionization time-of-flight mass spectrometry initially identified *Trichosporon inkin* fungi. However, phylogenetic analysis of intergenic spacer region 1 and whole-genome sequences revealed the genetically distinct species *T. austroamericanum*. Analysis of core genome and mitogenome from 6 patient isolates and 1 environmental isolate revealed substantial genetic diversity among *T. austroamericanum* strains, indicating a polyclonal outbreak. Furthermore, the mitochondrial genome emerged as a potential marker for intraspecies differentiation, which potentially could aid in epidemiologic investigations. Identified in 2024 but potentially underestimated, *T. austroamericanum* has since been reported in case clusters from hospital settings in France, highlighting the need for accurate fungal identification and suggesting previously identified *T. inkin* cases should be re-evaluated for *T. austroamericanum*. Clinical *T. austroamericanum* is emerging in hospital settings and should be included in the differential diagnosis of fungal infections.

Fungi belonging to various species of the genus *Trichosporon* can cause a wide range of infections, from superficial skin damage to serious systemic infections in immunocompromised persons, who can have high mortality rates (*1*). *Trichosporon*, which belongs to the Basidiomycota family, includes ≈20 species that are pathogenic to humans (*2*). Although *Cutaneotrichosporon cutaneum* and *T. asahii* are the predominant Basidiomycota species, new species have increasingly been described in human pathology since 2002 (*2*).

In 2024, a new Basidiomycota species, *T. austroamericanum*, was identified in a urine sample from a kidney transplant recipient in Brazil (*3*); the species was then detected in several clinical samples from France, South America, and Asia. *T. austroamericanum* and *T. inkin* have been confounded in the past before the species were distinguished through phylogenetic analysis of the intergenic spacer (IGS) 1 region and amplified fragment length polymorphism fingerprinting.

Ubiquitous in the environment, some *Trichosporon* species have been isolated from soil, leaf mold, and decayed wood (*4*). The environmental reservoirs of *T. inkin* and *T. austroamericanum* remain unknown. Moreover, emergence of hospital trichosporonosis cases caused by *T. asahii* have been described (*5*), highlighting hospital sources during epidemiologic and environmental investigations. Thus, *Trichosporon* species should not be overlooked in the hospital environment.

During July 2022–June 2024, the kidney transplant department of Marseille University Hospital, Marseille, France, recorded 4 cases of invasive fungal infection. Whole-genome sequencing (WGS) was initially performed on the first 3 reported cases by using matrix-assisted laser desorption/ionization time-of-flight (MALDI-TOF) mass spectrometry, which identified the agent as *T. inkin*. Further investigation using other molecular methods revealed *T. austroamericanum* fungi as the causative agent. Because of the rarity of the species, we suspected a putative common source. Moreover, 2 other cases subsequently were diagnosed in patients admitted in cardiology and gastrointestinal surgery centers in 2 other hospitals of our institution. We investigated the sudden upsurge of trichosporonosis cases through WGS and phylogenetic analysis.

## Materials and Methods

We prospectively identified patients over a 2-year period from 3 different sites of Marseille University Hospital system, referred to as hospitals A, B, and C. Our study included all patients with associated clinical signs of infection from whom *T. austroamericanum* fungi was isolated from sterile sites.

We collected retrospective medical data for each patient, when available, by using HOSPILINK DPI version 5.11.3P10.8.3 (Axigate Link, https://axigatelink.com) and processed data in an anonymized Excel 2013 file (Microsoft, https://www.microsoft.com). Data included demographic information, exposure factors, underlying diseases, and clinical signs and symptoms. 

### Inclusion and Ethics

We anonymized all identified *T. austroamericanum* strains recovered from clinical samples and stored them in Cryosysteme Protect bead tubes (Dutscher, https://www.dutscher.com) at −20°C. The hospital strain bank assigned a unique number to each sample: L0221, L0385, L0399, L0419, L0445, and L0458. The hospital routinely performed antifungal susceptibility testing by using the Sensititre YeastOne microdilution method (Thermo Fisher Scientific, https://www.thermofisher.com), according to the manufacturer’s instructions.

This study was reviewed and approved by the Ethics Committee of Assistance Publique Hôpitaux de Marseille (approval no. CSE24-49) and the Assistance-Publique-Hôpitaux de Marseille Health Data Access Portal (approval no. PADS24-275). Patients were informed of the research and their nonopposition to the use of their data was collected. In accordance with those committees and current regulations, written informed consent was not required. All potentially identifying information was removed in compliance with International Committee of Medical Journal Editors guidelines.

### Mycologic Investigation

From identified patients, we collected cryptococcal antigen in serum by using Cryptococcal Antigen Lateral Flow Assay (IMMY, https://www.immy.com) and Platelia *Aspergillus* Antigen (Bio-Rad Laboratories, https://www.bio-rad.com). In addition, the hospital’s infection control team initiated an environmental investigation in 2023, which they continued in 2024 until the last infected patient was identified. The investigation focused on the rooms and units where the initial kidney transplant patients had been under care from the date of admission to discharge, including radiology, the intensive care unit (ICU), the urological surgery operating theater, and the dialysis unit. The team also investigated the nephrology unit, where patients were hospitalized for a week after kidney transplantation. The team collected air samples by using AESAP1075 Sampl’air Lite microbiological air sampler (AES Laboratories, https://www.chemeurope.com), collecting 330 liters on Sabouraud dextrose agar (SDA). The team collected water samples from faucets or showers in patients’ rooms in 250-mL bottles containing sodium thiosulfate. The team also collected surface samples by using eSwab Liquid Amies Elution Swabs (Copan, https://www.copangroup.com). 

### Next-Generation Sequencing

We performed DNA extraction on pure subculture from SDA supplemented with gentamicin and chloramphenicol (Bio-Rad), using previously described methods (*6*). We sequenced genomic DNA by using a paired-end strategy. We barcoded and prepared samples by using the COVIDseq Test sample prep kit (Illumina, https://www.illumina.com) adapted for fungi, in which the tagmentation step fragmented and tagged the DNA. We used limited cycle PCR amplification (12 cycles) to complete the tag adapters and introduce dual-index barcodes. After purification on ITB beads (Illumina), we normalized libraries to the same molarity, then pooled those into a single library for sequencing on the NovaSeq 6000 (Illumina). We loaded the pooled single-strand library onto the reagent cartridge and then onto the instrument along with the flow cell. We conducted automated cluster generation and paired-end sequencing of dual index reads in a single 25-hour run of 2 × 150 bp.

We also performed nanopore sequencing on individually sequenced genomic DNA by using the PromethION 2 Solo or GridION and the LSK109 Ligation Kit on a FLO-PRO002 flow cell (all Oxford Nanopore Technologies, https://nanoporetech.com). The end-prep step fixes specific nucleotides to use for adapter ligation, after which we purified the DNA on magnetic beads (CleanNA, https://www.cleanna.com). We activated the flow cell by adding a flush buffer and a tether from Flow Cell Priming Kit EXP-FLP002 (Oxford Nanopore Technologies). We then loaded the libraries onto the flow cell for a 72-hour run.

### Bioinformatics

We converted Illumina binary base call (BCL) files into fastq files by using bclconverter version 4.2.4 (Illumina). We used Trimmomatic version 0.39 (*7*) to trim reads to a minimum Phred quality of 33 and minimum read length of 36 bp. We trimmed nanopore raw reads by using ProwlerTrimmer (*8*), a Phred quality of 20–35, and minimum read length of 1,000 bp.

We obtained complete genomes from mixed de novo assembly of Illumina and nanopore reads by using Unicycler version 0.4.4 (*9*). We removed contigs <1,000 bp in length. We determined GC content and evaluated contamination by using ContEst16S (EZBio https://www.ezbiocloud.net). We assessed assembly quality by using BUSCO version 5.7.1 and the fungi_odb10 database (*10*).

We used the Burrows-Wheeler aligner (Galaxy version 2.3 plus galaxy0; https://usegalaxy.eu) (*12*) to map filtered reads to the reference genome CBS 17435 (*11*) from the CBS culture collection (https://wi.knaw.nl/fungal_table) hosted by the Westerdijk Fungal Biodiversity Institute (Utrecht, the Netherlands). We generated consensus sequences by using iVar consensus (Galaxy version 1.4.4 plus galaxy0) (*13*), with depth coverage of >10 reads and base quality of Q20. We aligned the resulting sequences by using MAFFT (Galaxy version 7.526 plus galaxy2) (*14*).

We calculated pairwise single-nucleotide polymorphism (SNP) distances by using SNP distance matrix (Galaxy version 7.526 plus galaxy2) and identified SNPs by using the Find SNP Sites command (Galaxy version 2.5.1 plus galaxy0) (*15*). We aligned concatenated SNP lists and analyzed with IQ-TREE version 2.4.0 (http://www.iqtree.org) plus galaxy1 to generate a maximum-likelihood phylogenetic tree, which we visualized on iTOL (https://itol.embl.de). We calculated genome coverage with a custom Python script (Python Software Foundation, https://www.python.org).

We isolated mitochondrial genomes into a single circular contig ≈40-kb in size and aligned contigs by using mauve version 2.4.0 (*16*). We oriented contigs from the cytochrome c oxidase 1 start codon. When necessary, we generated reverse complements using an online tool (https://reverse-complement.com/terms.html), then annotated genomes by using GeSeq (https://chlorobox.mpimp-golm.mpg.de/geseq.html).

We mapped trimmed Illumina reads against the reference IGS1 sequence (CBS 17435) using Burrows-Wheeler aligner (*12*) and obtained consensus sequences by using sam2consensus (https://github.com/edgardomortiz/sam2consensus). We used MAFFT (https://mafft.cbrc.jp/alignment/server/index.html) (*13*) to align sequences and performed subsequent phylogenetic analysis by using IQ-TREE with 1,000 bootstraps (*17*).

### Maximum-Likelihood Core-Genome Phylogenetic Analysis 

We constructed a core-genome tree by using the Galaxy platform. We masked repeats with RepeatMasker (Galaxy version 4.1.5 plus galaxy0) and annotated the assemblies by using the Maker annotation pipeline (*18*) with the Augustus predefined prediction model *Cryptococcus neoformans gattii* (Galaxy version 2.31.11 plus galaxy2). We derived the aligned core-genome from the resulting annotated general feature format files by using Roary in Galaxy version 3.13.0 plus galaxy3, then collected SNPs by using the Find SNP Sites tool (Galaxy Version 2.5.1 plus galaxy0). We used those SNPs to build the maximum-likelihood tree on IQ-TREE version 2.3.6 (built August 4, 2024) (*17*), under ModelFinder (*19*). We visualized trees by using the iTOL platform.

## Results

### Patient Data

By June 2024, a total of 6 patients with *T. austroamericanum* infection had been identified (Table 1). The patients’ median age was 65.5 (range 55–83) years; 3 (50%) were male and 3 (50%) were female. Four patients were kidney transplant recipients who had clinical signs of an infection 2–4 months after transplantation; all 4 had scar dehiscence and subcutaneous abscesses, suggesting that the scar was the portal of entry. 

**Table 1:** Characteristics of patients from investigation of *Trichosporon austroamericanum* infections among hospitalized patients, France, 2022–2024*

Characteristics	Sample no.					
	L0221	L0385	L0399	L0419	L0445	L0458
Patient location						
Hospital	A	A	A	B	C	A
Hospital ward						
Kidney transplantation unit	Y	Y	Y	N	N	Y
Cardiac surgery	N	N	N	Y	N	N
Gastrointestinal surgery	N	N	N	N	Y	N
Clinical signs at time of positive culture						
Fever	Y	Y	N	N	N	N
Asthenia	Y	Y	N	Y	Y	N
Dyspnea	N	Y	N	N	Y	N
Digestive symptoms	N	N	N	N	Y	N
Scar dehiscence	Y	Y	Y	NA	NA	Y
Biology at time of positive culture						
Creatinine, µmol/L	271	128	103	177	80.7	216
C-reactive protein, mg/L	86	73	43	22	NP	1.3
Positive culture results						
Date of first positive culture	2022 Jul	2023 Apr	2023 Jun	2023 Dec	2024 Apr	2024 Jun
Days after transplantation	71	78	135	NA	NA	115
No. positive samples	4	11	1	5	3	1
Blood	0	11	0	5	0	0
Scar swab	3	0	0	0	0	0
Abscess aspiration	1	0	1	0	0	1
Respiratory sample	0	0	0	0	3	0
Microbiology						
Cryptococcal antigen	Positive	Positive	Positive	Negative	NP	Negative
Titer	160	5,120	20	–	–	–
*Aspergillus* antigen	Negative	NP	NP	NP	Negative	NP
Index	0.04	–	–	–	0.13	–
Outcome						
Death within 90 d	N	N	N	Y	Y	N
No. days after first positive culture	NA	NA	NA	30	30	NA
Treatment						
Posaconazole	N	Y	N	N	N	N
Voriconazole	Y	Y	Y	N	N	Y
Amphotericin B	N	Y	N	N	N	N
Surgical debridement	Y	Y	Y	NA	N	Y
Immunosuppressant reduction	Y	Y	N	NA	NA	Y

*NA, not applicable; NP, not performed; –, no data.

The 2 other patients were immunocompetent. One (sample no. L0419) had been hospitalized in the cardiology department and was referred for a nephrology consultation during the time the outbreak was occurring. That patient had not undergone recent surgery. The other patient (sample no. L0445) experienced shock and acute respiratory distress syndrome after gastrointestinal surgery.

Of the 25 *T. austroamericanum*–positive samples, 16 (64%) were blood cultures from 2 patients, 3 were scar swab samples from 1 patient, 3 were abscess aspiration samples from 3 patients, and 3 were respiratory samples (2 bronchial aspirate and 1 bronchoalveolar lavage fluid) from 1 patient. All *T. austroamericanum* isolates exhibited similar antifungal susceptibility profiles (Table 2), and we noted no significant difference related to date of isolate collection.

**Table 2:** Antifungal susceptibility testing of isolates from investigation of *Trichosporon austroamericanum* infections among hospitalized patients, France, 2022–2024*

Strain no.	MIC per antifungal agent, µg/mL								
	AMB	5-FC	FCZ	ITC	VRC	POS	CAS	MCF	AND
L0221	1	64	0.5	0.12	0.03	0.12	8	8	8
L0385	0.25	32	0.5	0.06	0.015	0.06	>8	>8	>8
L0399	0.25	16	1	0.06	0.015	0.12	>8	>8	>8
L0419	0.12	>64	1	0.06	0.015	0.06	>8	>8	>8
L0445	2	>64	1	0.12	0.03	0.25	>8	>8	>8
L0458	0.5	32	1	0.12	0.03	0.12	8	8	8

*AMB, amphotericin B; ANI, anidulafungin; CAS, caspofungin; FCZ, fluconazole; ITC, itraconazole; MIF, micafungin; VRC, voriconazole; POS, posaconazole; 5-FC, 5-Fluorocytosine.

### Mycological Investigation

The infection control team conducted an environmental survey in hospital A during April 20, 2023–July 19, 2024. In total, they collected 145 surface samples, 33 air samples, and 17 water samples in the urological surgery operating theater and the following patient units: the nephrology ICU, the kidney transplantation unit, the nephrology daycare, the radiology unit, and the dialysis unit. Samples were mainly collected from patient rooms, medical device storage area, and the decontamination room. All 195 samples were negative for *T. austroamericanum*. However, a *T. austroamericanum* strain was isolated from an air sample taken as part of a routine surveillance in a pediatric ICU of hospital B.

### Next-Generation Sequencing

#### Multilocus Sequence Typing

**Figure 1:**
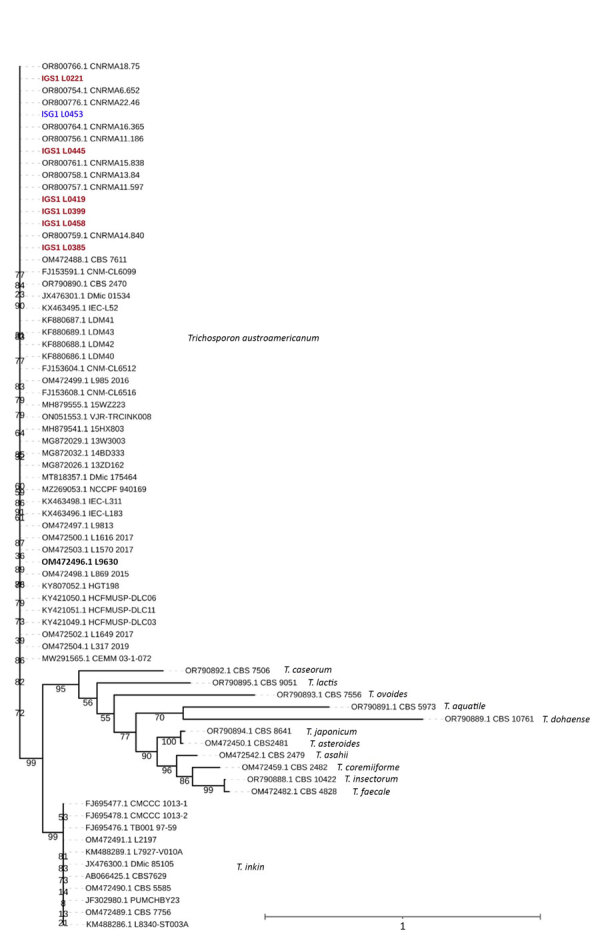
Maximum-likelihood phylogenetic tree of IGS1 sequences from study of *Trichosporon austroamericanum* infections among hospitalized patients, France, 2022–2024. The tree includes strains isolated from 6 patients (red font) and 1 environmental sample (L0453, blue font), mapped against *T. austroamericanum* and related species from GenBank (https://www.ncbi.nlm.nih.gov/genbank) and the CBS culture collection (https://wi.knaw.nl/fungal_table). Bold font indicates reference strain CBS 17435. The clustering confirms that the patient and environmental strains belong to *T. austroamericanum* and form a distinct clade. The tree also shows the relationships between other *Trichosporon* species, such as *T. inkin*, *T. caseorum*, and *T. ovoides*, and other *T. austroamericanum* reference strains from the National Reference Center for Invasive Mycoses and Antifungals at Institut Pasteur (https://www.pasteur.fr). Bootstrap values are indicated at the nodes. Scale bar indicates nucleotide substitutions per site. IGS, intergenic spacer region.

We initially performed WGS on the first 3 reported cases, which MALDI-TOF mass spectrometry had identified as *T. inkin*. To investigate genetic variations, we applied a multilocus sequence typing approach. We mapped sequences of *cytb*, *rpb1/2*, and *tef1* genes and the SSU and D1/D2 regions against the reference genome of *T. inkin* (GenBank accession no. MT801082). Although the sequences were identical among the isolates, notable differences from *T. inkin* emerged among isolates from those 3 cases. In addition, we observed high divergence between the strains from our investigation and the *T. inkin* reference mitochondrial genome and IGS1 marker, characterized by numerous point mutations, deletions, and insertions (Appendix Figure 1). The consistent mutation patterns across the strains, along with their divergence from *T. inkin*, strongly supported the classification of the isolates as a distinct taxonomic entity. That preliminary analysis served as the starting point for further investigation into the IGS1 region of the 6 patient-derived isolates. Phylogenetic analysis, which included 3 reference sequences provided by the National Reference Center for Invasive Mycoses and Antifungals (CNRMA) at Institut Pasteur (https://www.pasteur.fr), confirmed that the 7 strains (6 patient and 1 environmental) belonged to *T. austroamericanum* species (Figure 1).

#### WGS Typing

We conducted WGS typing to assess the clonal nature of the *T. austroamericanum* outbreak. Of the 7 genomes, 6 demonstrated excellent assembly quality and had contigs ranging from 12 to 31 (Table 3). Those assemblies showed consistent genome lengths of 20.8 to 21 Mb, and GC content was close to 61.31%. The BUSCO scores were all >96% (range 96.5%–97.4%), indicating highly complete and accurate assemblies. However, the assembly for strain L0445 stood out because it had a much higher number of contigs (1,051), a larger genome size of 26 Mb, and a higher GC content of 62.59%. That assembly also showed contamination with bacterial *Achromobacter xylosoxidans* 16S sequence, so we excluded it from further analyses.

**Table 3:** Characteristics of isolates from investigation of *Trichosporon austroamericanum* infections among hospitalized patients, France, 2022–2024

Characteristics	Isolate no.						
L0221	L0385	L0399	L0419	L0445	L0453	L0458
Contigs	14	12	16	13	1,051	31	24
Length, bp	20,873,347	20,931,201	21,007,957	20,906,681	26,088,532	20,871,584	20,893,304
% GC	61.3	61.31	61.31	61.31	62.59	61.31	61.31
Contamination	N	N	N	N	Y	N	N
BUSCO C-score, %	96.50	96.90	97.40	96.60	96.10	96.90	96.90

**Figure 2:**
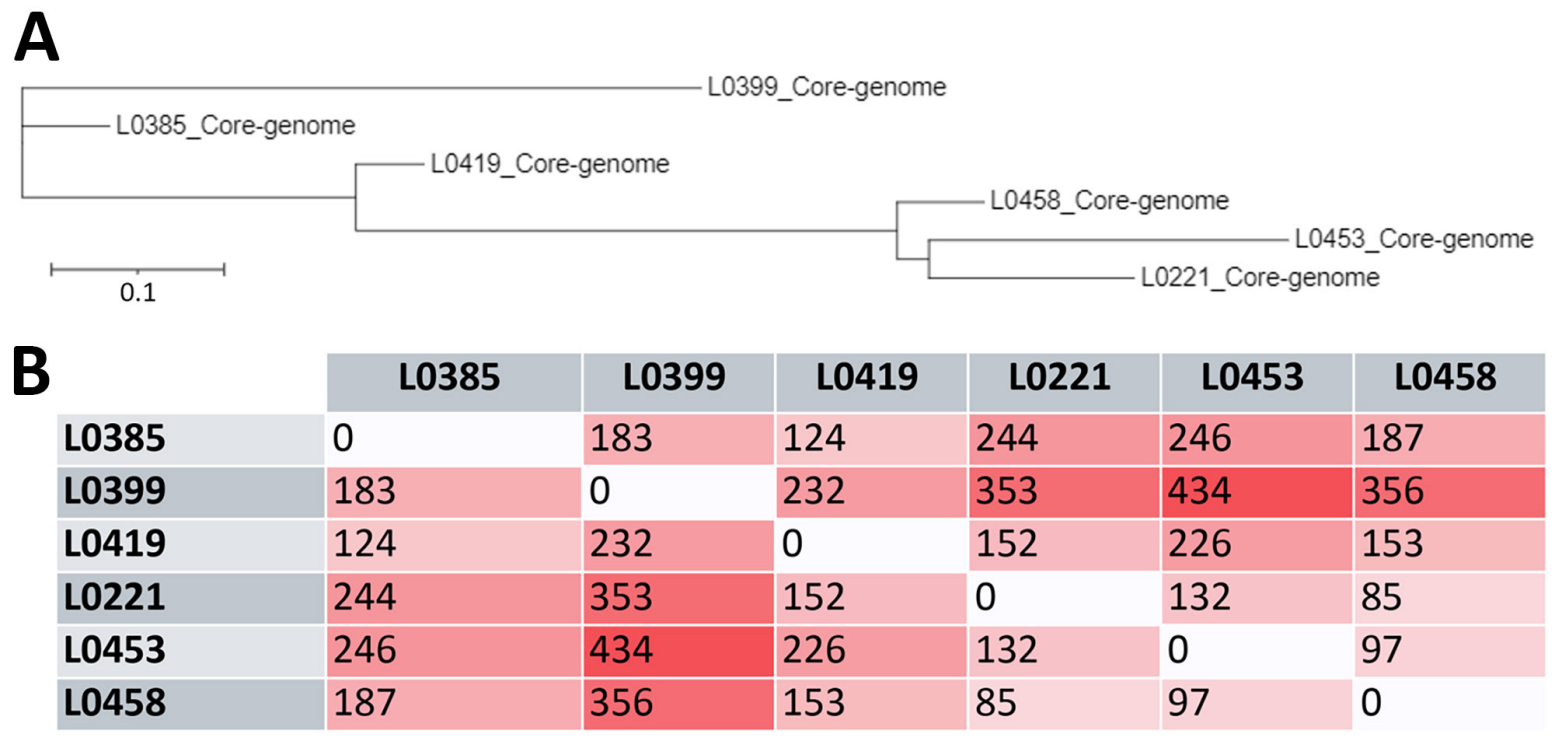
Core-genome phylogenetic relationships and single-nucleotide polymorphism (SNP) distance matrix from study of *Trichosporon austroamericanum* infections among hospitalized patients, France, 2022–2024. A) Phylogenetic tree based on the core genome of the analyzed strains showing the evolutionary relationship between strains. Distance between branches reflects the degree of genetic divergence on the basis of SNP variations in the core genome. Scale bar indicates nucleotide substitutions per site. B) Core-genome SNP distance matrix. Each value represents the number of SNPs that differ between genome pairs. Darker shades indicate greater distance.

The core genome consisting of 11,957 genes enabled construction of a phylogenetic tree from the extracted SNPs, which formed sequences with 542 nt sites and 196 parsimony-informative positions (Figure 2, panel A). Of the 542 sites, we observed 129 distinct site patterns, indicating a high level of sequence variation across the strains. Strains L0453 and L0221, separated by only 85 SNPs (Figure 2, panel B), were the closest in the tree. L0453, from the ICU air duct, also appeared near L0458, with a difference of 97 SNPs. In addition, we identified 7,668 shell genes, representing genes shared by a subset of strains, indicating genetic diversity beyond the core genome.

#### Whole-Genome SNP Analysis

We performed whole-genome SNP calling by mapping the Illumina reads of our strains against *T. austroamericanum* reference genome CBS 17435 (*11*). That method enabled us to include strain L0445, for which high-quality mapping data was available. Read mapping demonstrated high genome coverage quality across all 8 chromosomes and strict coverage exceeding 97% for all strains (minimum coverage 97.30% +SD 0.00368%) and reaching up to 99.98% (+SD 0.00043%).

**Figure 3:**
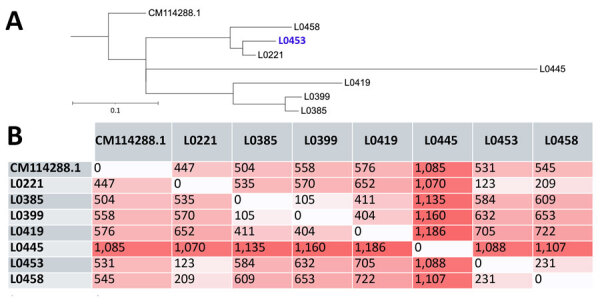
Whole-genome single-nucleotide polymorphism (SNP)–based phylogeny and distance matrix from study of *Trichosporon austroamericanum* infections among hospitalized patients, France, 2022–2024. A) Whole-genome phylogenetic tree of the analyzed strains. Blue font indicates reference strain; blue font indicates environmental strain. Scale bar indicates nucleotide substitutions per site. B) Whole-genome SNP distance matrix. Each value represents the number of SNPs that differ between genome pairs. Darker shades indicate greater distance.

The resulting whole-genome SNP distance matrix revealed patterns consistent with the core genome analysis (Figure 3, panel A). Distances between the CBS 17435 reference and the clinical strains ranged from 447 to 1,085 SNPs. The smallest pairwise distance was 105 SNPs between L0385 and L0399, which were 2 isolates collected within 3 months of each other from the same hospital, supporting their close genetic relationship. The second closest group consisted of L0221, L0453, and L0458, which clustered together despite being collected in different years (Table 1). Most other pairwise distances ranged from ≈400 to >1,100 SNPs, including between L0445 and the rest of the isolates (≈1,070–1,186 SNPs), suggesting that L0445 is genetically more distant, consistent with its location at a hospital site geographically distant from the others.

#### Mitochondrial Genome

As a complementary approach, we analyzed the mitochondrial genome to assess its potential for discriminating between strains. We used mapping to obtain mitochondrial genomes of ≈44 kb from 3 *T. austroamericanum* reference strains from the CNRMA and the L0445 strain, then generated genomes of other 6 genomes through de novo assembly (Appendix Figure 2). Of note, synteny was identical between the de novo assembled mitogenomes.

**Figure 4:**
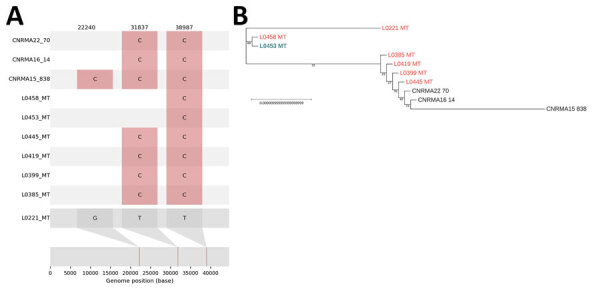
Single-nucleotide polymorphism (SNP) map and phylogenetic analysis of mitogenomes from investigation of *Trichosporon austroamericanum* infections among hospitalized patients, France, 2022–2024. A) SNP map of mitochondrial genomes from *T. austroamericanum* isolates. The SNP alignment highlights 3 key positions in the mitochondrial genome (at positions 22240 in the *cox3* gene, 31837 in the *apocytochrome b* gene, and 39897 in the *trnL* [tRNA-Leu] gene). B) Phylogenetic placement of clinical isolates (red font), the environmental strain (blue font), and strains from the National Reference Centre for Invasive Mycoses and Antifungals at Institut Pasteur (https://www.pasteur.fr). Scale bar indicates nucleotide substitutions per site.

The alignment of those mitochondrial genomes against the de novo assembled L0221 sequence revealed 4 distinct mutational profiles, including 3 among Marseille strains (Figure 4). Those profiles included 3 key mutated positions in the mitochondrial genome of the *cox3 *(at position 22240), *apocytochrome b* (at position 31837), and *trnL* (tRNA-Leu) (at position 39897) genes. The mutations provide clear genetic distinctions among the strains. For instance, strain CNRMA15 exhibited a unique mutation, C at position 22240, whereas other strains, such as L0221, have a G at that position (Figure 4, panel A). That analysis underscores the value of mitochondrial genome sequencing for identifying and distinguishing between *T. austroamericanum* strains involved in this outbreak and future outbreaks. 

The phylogenetic placement of mitochondrial genomes showed 4 distinct clades. L0221 and CNRMA15 formed a separate branch because of their unique SNP profiles. L0458 and L0453 were closely related, as seen in the core-genome analysis. Finally, L0385, L0419, L0399, L0445, CNRMA22, and CNRMA16 cluster together, and CNRMA 15 branches off the others (Figure 4, panel B).

## Discussion

With ≈20 pathogenic species, *Trichosporon* spp. are the second leading cause of Basidiomycota infections in humans (*2*). Although rare, *Trichosporon* infections in solid organ transplant patients have previously been reported and should be considered in cases of breakthrough infection or echinocandin therapeutic failure (*20*). In this case series, 67% of patients with invasive *T. austroamericanum* infection were kidney transplant recipients. For all kidney transplant recipients, the starting point was infection at the graft scar that occurred within 2–4 months of transplantation, suggesting a common source of contamination at the time of surgery. Of note, 2 patients in 2 different hospitals also had invasive *T. austroamericanum* infections, thus challenging the common source assumption. However, we did not observe any major changes in the antifungal susceptibility profiles that would have contradicted the idea of a common origin or that would have reflected an evolution of the strain over time (Table 2).

**Figure 5:**
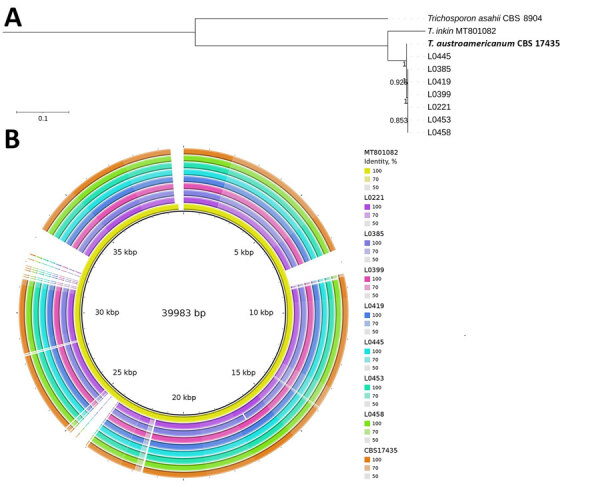
Maximum-likelihood phylogenetic tree and BLAST ring of isolates from a study of *Trichosporon austroamericanum* infections among hospitalized patients, France, 2022–2024. A) Maximum-likelihood phylogenetic tree of the mitochondrial genome, showing the relationship between *T. inkin*, *T. asahii*, *T. austroamericanum* reference strain (bold font), and *T. austroamericanum* isolates from this study. Scale bar indicates nucleotide substitutions per site. B) Mitogenome alignment of *T. austroamericanum* isolates against *T. inkin* (MT801082), obtained by using BLAST Ring Image Generator (https://github.com/happykhan/BRIG).

*T. austroamericanum* was initially described in May 2024 by E.C. Francisco et al. (*3*). The first case we report appeared in July 2022, but because our database did not yet contain *T. austroamericanum* reference strains, the *Trichosporon* species were initially identified by MALDI-TOF mass spectrometry as *T. inkin*, a closely linked species. In the absence of a satisfactory reference genome, we focused on the *T. inkin* mitochondrial genome, the only genome available and well described in the literature (*21*). We observed considerable differences between the genomes of our strains, and the reference mitochondrial genome of *T. inkin* (Figure 5). The *T. austroamericanum* species described by Francisco et al. reinforced our analyses. 

IGS1 is the region of interest for discriminating between different *Trichosporon* species (*3*,*22*,*23*). Comparing the rRNA IGS1 region nucleotide sequences between our strains with the CNRMA *T. austroamericanum* strains enabled us to confirm the result of mitochondrial genome analysis and revealed that our hospital was facing an emergence of *T. austroamericanum*. Moreover, the mitochondrial genome showed 3 different profiles within the *T. austroamericanum* strains isolated in Marseille and 3 key mutation positions. The appearance of spontaneous mutations has been described in the mitochondrial genome of certain phytopathogenic species, probably resulting from replication errors and the presence of mobile genetic elements (*24*).

We investigated a potential common origin through genomic analysis, as previously described (*25*). We produced complete genomes of *T. austroamericanum* by de novo WGS. Drawing inspiration from other epidemic investigations (*26*,*27*), we chose to target conserved sequences for a core-genome SNP typing approach (Figure 2). After publication of the *T. austroamericanum* CBS 17435 reference genome (*11*), we conducted a WGS and SNP analysis. Whole-genome SNP distances between the CBS 17435 reference strain from Brazil and our strains ranged from 447 to 1,186 SNPs. Of note, that range of differences was of the same order of magnitude as what we observed among the individual strains in our study, supporting the hypothesis of a polyclonal outbreak. Such an approach had already been used to identify clusters of 2 other basidiomycetes, *T. asahii* and *Rhodotorula* spp. (*28*,*29*). 

No consensus regarding SNPs among fungal species exists because many strains are needed to study mutation rates, which is not always possible for rarer species. Thus, the cutoff point to determine whether strains are related seems to be set at <15 SNPs for *Candida auris* and 1,200 SNPs for other species, such as *Rhodotorula mucilaginosa* (*28*,*30*). Nevertheless, some strains in our study were genetically much closer. L0385 and L0399 differed by only 105 SNPs and were isolated from the same site within a short interval, suggesting a recent common origin or persistence from an environmental reservoir (Figure 3).

Phylogenetic analysis of the mitochondrial genome, core genome, and whole genome showed similar results, highlighting the benefits of those phylogenetic analyses in epidemiologic fungal investigations. Although the core-genome analysis was based on only a limited number (11,957) of genes, it enabled distinguishing the species and obtaining a phylogenetic placement like that from whole-genome analysis.

The hospital’s infection control team also conducted an environmental survey to identify a source of contamination, but investigations in the kidney transplant and nephrology departments did not reveal any common source of contamination. Investigations elsewhere have isolated *Trichosporon* species from the hospital environment. One published study identified 53 patients with positive *T. asahii* cultures in a hospital in Jamaica, 4 of whom were hospitalized in an ICU and had invasive *T. asahii* infection (*5*). After an environmental investigation in that hospital, 10 surface swab samples from the patient rooms showed *T. asahii*, including samples from drawers, bed rails, faucets, and sinks (*5*). In our investigation, *T. austroamericanum* was isolated (no. L0453) from an air sample from a pediatric ICU at hospital B, but no infection or colonization with that microorganism was noted among patients from that unit. We found no epidemiologic link between that unit and the reported clinical case-patients, all of whom were treated in other hospitals. The lack of an epidemiologic link suggests that *T. austroamericanum* was part of the hospital environment. In our study, the whole-genome SNP analyses showed 123–1,088 SNPs difference between the clinical strains and the environmental strain. However, that finding does not exclude the possibility of multiple clonally unrelated strains circulating and acquired within hospital environmental reservoir. 

The results of this study demonstrate that core-genome analysis can effectively differentiate *T. austroamericanum* strains, revealing a polymorphic species. Furthermore, the mitochondrial genome shows strong potential as an excellent marker for intraspecies differentiation. That approach is particularly valuable in the absence of available annotated genomes for *T. austroamericanum* and was supplemented and reinforced by the release of the reference genome CBS 17435 (*11*).

In conclusion, our findings suggest that certain previously deposited sequences identified as *T. inkin* should be re-evaluated for *T. austroamericanum* to account for the emergence of this newly described species. In addition, this study underscores the need for vigilance regarding *T. austroamericanum* infections, including the potential for nosocomial involvement. 

Appendix Additional information on *Trichosporon austroamericanum* infections among hospitalized patients, France, 2022–2024.
