# Supplementary material for: Trichosporon austroamericanum Infections among Hospitalized Patients, France, 2022–2024
Source: Emerg Infect Dis. 2025 Nov;31(11):2080–90. doi: 10.3201/eid3111.250503 (PMC12704532; doi:10.3201/eid3111.250503)
Supplement: Appendix — Additional information on Trichosporon austroamericanum infections among hospitalized patients, France, 2022–2024. [file 25-0503-Techapp-s1.pdf]

Article DOI: https://doi.org/10.3201/eid3111.250503

EID cannot ensure accessibility for supplementary materials supplied by authors. Readers who have difficulty accessing supplementary content should contact the authors for assistance.

Trichosporon austroamericanum Infections among Hospitalized Patients, France, 2022–2024

Appendix

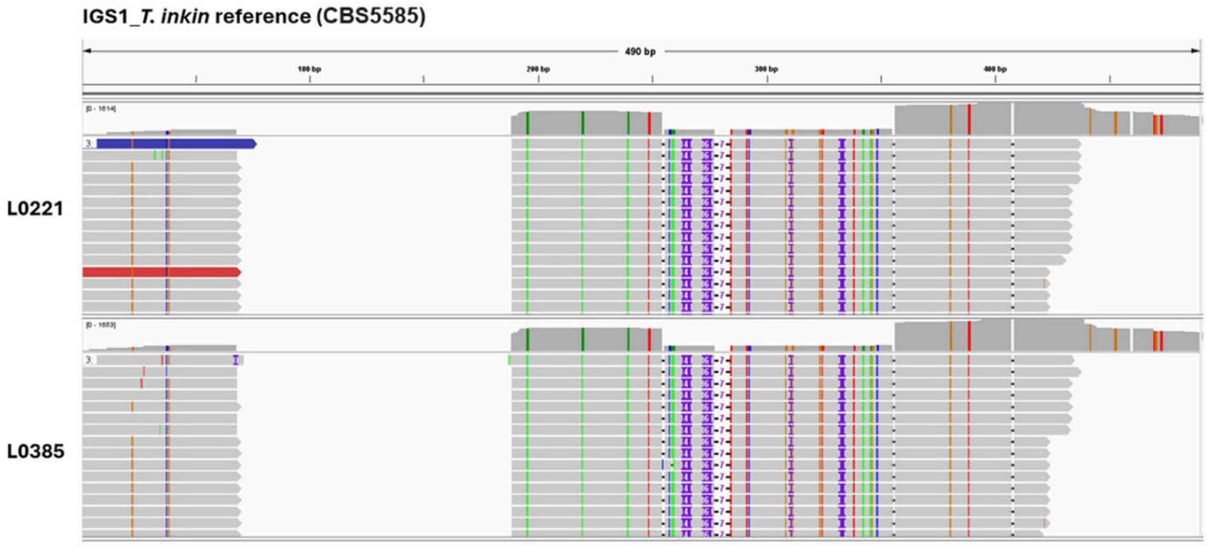

Appendix Figure 1. Alignment of IGS1 Sequences of *Trichosporon* Strains L0221 and L0385 against *Trichosporon inkin* reference (CBS5585). The horizontal axis represents the length of the IGS1 region, with nucleotide variations displayed as colored bars. Differences between the sequences and the reference are indicated by colored lines, with mismatches, insertions in purple, and deletions highlighted with a dash.

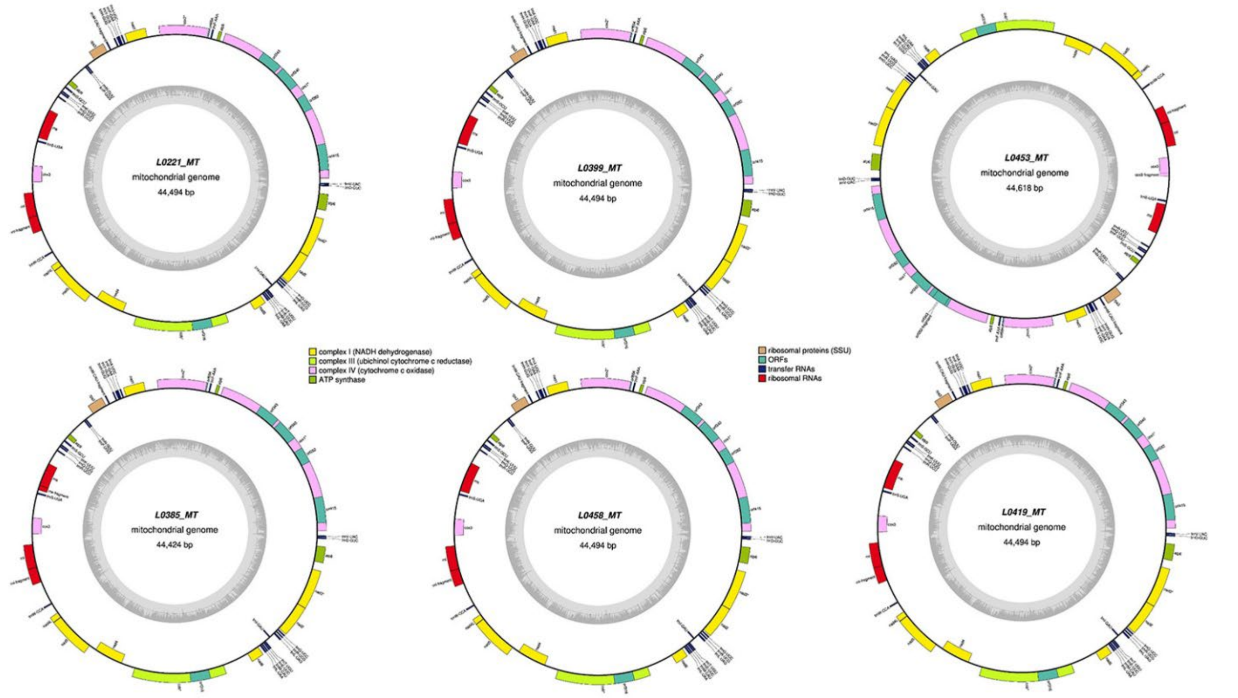

Appendix Figure 2. Mitochondrial genome maps of the seven *T. austroamericanum* strains assembled de novo. Circular representations and annotation of the six mitogenomes obtained by de novo assembly, for strains L0221, L0385, L0399, L0419, L0453 and L0458. The outer depicts annotated genes, color-coded according to their function: protein-coding genes (yellow), rRNA genes (green), tRNA genes (pink), and non-coding regions (red). The size of each genome is indicated in the center, ranging between 44,424 to 44,494 bp, obtained in one contig from the de novo assemblies. Differences in the arrangement and number of certain SNPs (to be completed in the figure) and genomic features are noted, highlighting potential mitochondrial genotypic diversity among the strains.
